# Increased arbuscular mycorrhizal fungal colonization reduces yield loss of rice (*Oryza sativa* L.) under drought

**DOI:** 10.1007/s00572-020-00953-z

**Published:** 2020-04-15

**Authors:** Anupol Chareesri, Gerlinde B. De Deyn, Lidiya Sergeeva, Anan Polthanee, Thomas W. Kuyper

**Affiliations:** 1 Department of Environmental Sciences, Soil Biology Group, Wageningen University & Research, P.O. Box 47, 6700 AA Wageningen, The Netherlands; 2 Laboratory of Plant Physiology, Wageningen University & Research, Wageningen, The Netherlands; 3 Department of Plant Science and Agricultural Resources, Faculty of Agriculture, Khon Kaen University, Khon Kaen, Thailand

**Keywords:** Stomatal conductance, Chlorophyll fluorescence, Abscisic acid (ABA), Indole acetic acid (IAA)

## Abstract

Drought reduces the availability of soil water and the mobility of nutrients, thereby limiting the growth and productivity of rice. Under drought, arbuscular mycorrhizal fungi (AMF) increase P uptake and sustain rice growth. However, we lack knowledge of how the AMF symbiosis contributes to drought tolerance of rice. In the greenhouse, we investigated mechanisms of AMF symbiosis that confer drought tolerance, such as enhanced nutrient uptake, stomatal conductance, chlorophyll fluorescence, and hormonal balance (abscisic acid (ABA) and indole acetic acid (IAA)). Two greenhouse pot experiments comprised three factors in a full factorial design with two AMF treatments (low- and high-AMF colonization), two water treatments (well-watered and drought), and three rice varieties. Soil water potential was maintained at 0 kPa in the well-watered treatment. In the drought treatment, we reduced soil water potential to − 40 kPa in experiment 1 (Expt 1) and to − 80 kPa in experiment 2 (Expt 2). Drought reduced shoot and root dry biomass and grain yield of rice in both experiments. The reduction of grain yield was less with higher AMF colonization. Plants with higher AMF colonization showed higher leaf P concentrations than plants with lower colonization in Expt 1, but not in Expt 2. Plants with higher AMF colonization exhibited higher stomatal conductance and chlorophyll fluorescence than plants with lower colonization, especially under drought. Drought increased the levels of ABA and IAA, and AMF colonization also resulted in higher levels of IAA. The results suggest both nutrient-driven and plant hormone-driven pathways through which AMF confer drought tolerance to rice.

**Electronic supplementary material:**

The online version of this article (10.1007/s00572-020-00953-z) contains supplementary material, which is available to authorized users.

## Introduction

Rice (*Oryza sativa* L.) is a staple food feeding more than half of the world’s population. Demand for it is increasing due to an increase in the global population (FAO 2002). More than 75% of global rice production is from lowland rice cultivated under submerged conditions, and the remainder is from upland rice grown under non-submerged conditions (Maclean et al. 2013). Submerging rice paddy fields increases the availability of nutrients in the soil and limits the growth of weeds. However, this practice requires very large amounts of water (Haefele et al. 2008). Whereas, water can be sufficiently supplied in irrigated farming systems, water availability is a problem under rain-fed rice farming. Under those conditions, rainfall is the only water source; therefore, the productivity is highly dependent on the amount of rainfall. Less rainfall leads to water deficit in the soil, inducing drought, which can be a major constraint for producing rice.

Due to global climate change, drought likely will occur more frequently and more severely than in the past, causing problems for crop production in several regions of the world. Compared to other cereals, rice is particularly sensitive to drought. Drought affects growth and grain yield of rice by limiting water and nutrient availability, especially phosphorus (P) (Suriyagoda et al. 2014). In addition, drought reduces stomatal conductance as a mechanism to reduce water loss. However, lower stomatal conductance decreases gas exchange and photosynthesis efficiency and reduces yields (Lauteri et al. 2014).

The effects of drought on rice depend on timing and severity (Prasertsak and Fukai 1997). For instance, drought during vegetative stages has a smaller negative impact on yield than when it occurs during the panicle development stage (Boonjung and Fukai 1996). Drought reduces leaf expansion and delays maturation during the vegetative stage (Lilley and Fukai 1994). Drought can reduce rice yield by more than 60% when it occurs during the panicle development stage (Boonjung and Fukai 1996; Venuprasad et al. 2007). Growth and yield reduction can be mitigated by irrigation. However, such management is not practical for rain-fed farming. Hence, rain-fed farming needs other means to cope with drought. Some drought-tolerant rice varieties have been successfully developed through breeding, for instance, *Sahbhagi Dhan* in India, *Sahod Ulan* in the Philippines, *Sookha Dhan* in Nepal, and *IR64* in India (Dar et al. 2014). These varieties yield 0.8–1.2 t ha^−1^ more than drought-susceptible varieties under drought (Dar et al. 2014).

Plant phenotypic plasticity is important to cope with drought, ideally enabling plants to withstand drought without a yield penalty. Highly adaptable plants can respond to drought by producing more roots, reducing water loss via stomatal closure and early maturation (Jearakongman et al. 1995; Fukai and Cooper 1995). The changes of stomatal conductance under drought also can be related to plant growth hormones, for instance, abscisic acid (ABA) and indole-3-acetic acid (IAA). ABA is the hormone that inhibits shoot growth, especially under drought. Under drought, ABA will be produced in the shoot (Borghi et al. 2015) or transported from the root to the shoot (Ko and Helariutta 2017), inducing stomatal closure. Haider et al. (2018) found a significant increase in leaf ABA content of rice plants under drought. IAA is important for root and shoot development, and it has been reported that IAA can induce the establishment of arbuscular mycorrhizal fungi (AMF) (Lüdwig-Müller and Güther 2007; Fitze et al. 2005). The contribution of AMF to plant levels of IAA has not been well explored.

Apart from these mechanisms, some plants deal with drought through interaction with and assistance by soil micro-organisms. One major group of soil micro-organisms that play an important role in enhancing drought tolerance comprises AMF (Rodriguez and Redman 2008; Ruiz-Lozano and Aroca 2010; Ruiz-Sánchez et al. 2010; Ruiz-Lozano et al. 2016). The symbiosis of AMF and roots increases plant nutrient uptake under drought, especially phosphorus (P) uptake (Augé 2001). In addition, AMF can alter photosynthetic efficiency of plants under drought by maintaining stomatal conductance (Augé 2001; Querejeta et al. 2007; Augé et al. 2015; Ruiz-Lozano et al. 2016) and the efficiency of photosystem II (PS II) (Mirshad and Puthur 2016). Augé et al. (2015) reported that the stomatal conductance of mycorrhizal plants is 24% higher than that of non-mycorrhizal plants. The changes in stomatal conductance also may be related to the effects of AMF regulating plant hormones. According to Estrada-Luna and Davies (2003), the flux of ABA in the shoots of AMF plants is lower than in non-AMF plants, which also results in higher transpiration and leaf water potential.

There are only few studies on AMF symbiosis and rice. This may be because rice is mostly grown in waterlogged conditions, which usually inhibit AMF colonization. Nevertheless, the symbiosis of AMF and rice plants has been reported (Maiti et al. 1995; Wangiyana et al. 2006; Lumini et al. 2011; Watanarojanaporn et al. 2013; Vallino et al. 2014). For instance, the system of rice intensification promotes root colonization and diversity of AMF species in rice roots compared to conventional rain-fed rice cultivation systems (Watanarojanaporn et al. 2013). AMF are more abundant in low-input farming and under aerobic conditions than under partly anaerobic and submerged conditions (Lumini et al. 2011; Vallino et al. 2014). Hence, field management that promotes the functioning of AMF symbioses and possibly additional AMF inoculation could increase AMF colonization in rice. Increased AMF colonization may subsequently make rice more tolerant of drought, but the magnitude of this effect is still unknown and therefore needs to be investigated.

We investigated the contribution of AMF to the growth of six different rice varieties with different drought tolerances, under well-watered and drought conditions. Furthermore, we attempted to understand underlying mechanisms of mycorrhiza-enhanced drought tolerance, such as higher nutrient uptake, enhanced stomatal conductance, and elevated efficiency of PS II. We also studied the effects of AMF on the regulation of plant hormones in rice without and with drought. In this study, we included the measurement of ABA and IAA hormones. In order to add a level of realism with respect to field conditions, we compared plants with higher and lower colonization by AMF (through inoculum addition), rather than comparing plants with and without mycorrhizas. We hypothesized that

(i) Rice with higher levels of AMF colonization have higher uptake of N and P, and more biomass (shoot, root, grain yield) than plants with lower levels of colonization, and these AMF benefits are larger under drought than under well-watered conditions.
(ii) Rice with higher levels of AMF colonization have higher stomatal conductance and higher quantum yield of PS II (*F*_*v*_/*F*_*m*_) than plants with lower levels of colonization, and these mycorrhizal effects are larger under drought than under well-watered conditions.
(iii) Rice with higher levels of AMF colonization have lower leaf ABA and higher leaf IAA concentrations than plants with lower levels of AMF colonization.

## Material and methods

### Experimental setup

Two greenhouse pot experiments were conducted at Khon Kaen University, Thailand. Experiment 1 (Expt 1) was done in the rainy season (August 2016–January 2017) and experiment 2 (Expt 2) in the dry season (December 2017–May 2018). We did not control light and temperature in the greenhouse. Both experiments were set up as a randomized complete block design with three factors, comprising three rice varieties, two water treatments (well-watered and drought), and two AMF treatments (low- and high-AMF colonization, the latter treatment with inoculum addition) with eight replicates of each treatment.

### Soil

The soils used in this experiment were collected from a rice farm in Khon Kaen (16° 29′ 10.9′′ N and 102° 34′ 40.5′′ E), from the top layer of 0–15 cm. The soil properties were analyzed at the laboratory of the Agriculture Faculty, Khon Kaen University. Soil properties are shown in Supplementary Table 1. We used unsterilized soil in both experiments. The soil was sieved (2-mm sieve) and mixed homogeneously, and then used to fill the pots. Pot size was 0.0066 m^−3^ (top surface diameter = 0.24 m, bottom surface diameter = 0.17 m and height = 0.19 m). We filled the pots to 0.14 m depth, so each pot contained approximately 5.6 kg soil dry weight. The soil was saturated before planting rice seedlings. Pot weight of the saturated soil at 0 kPa was recorded for calculating the amount of water needed for rewetting the soil after imposing the drought treatment.

### Rice varieties

For Expt 1, we used three rice varieties, viz. Khao Dowk Mali 105 (KDML 105), RD6, and Surin 1 (SR1) (Supplementary Table 2). These three rice varieties are the most consumed and economically valuable varieties, especially KDML 105 which is also known as jasmine rice or pandan rice. These varieties are grown during the rainy season. KDML 105 is a lowland rice variety, sensitive to photoperiod, and drought-tolerant. RD6 is long-grain glutinous rice which was developed from KDML 105. It is also a lowland rice variety, photoperiod sensitive, and moderately drought-tolerant. SR1 was bred from IR61078 and IR46329-SRN-18-2-2-2, it is a long-grain lowland rice variety, not sensitive to photoperiod. SR1 is a highly drought-tolerant variety. Rice seeds were obtained from the Rice Seed Centre of Khon Kaen.

For Expt 2, we used three other lowland rice varieties that are not sensitive to photoperiod. The three rice varieties used were Chainart 1 (CNT1), RD22, and RD33 (Supplementary Table 2). We chose these varieties because the experiment was done during the dry season. These varieties are commonly grown during the dry season in irrigated regions. CNT1 is the most commonly grown rice variety. RD22 is a long-grain sticky rice that is more drought-sensitive than CNT1. RD33 is a variety obtained from breeding KDML 105 and IR70177-76-3-1. It is suggested to be grown in the rain-fed regions in the north and northeast of Thailand. For Expt 2, rice seeds were obtained from the Rice Research Centre of Sakonnakorn, Thailand.

### Arbuscular mycorrhizal fungi

The abundance of AMF in the soil was quantified through spore counting after wet-sieving. The wet-sieving was done by extracting 100 g of soil with water, and sieving through a stack of 45- and 35-μm sieves. Then, spores were counted under the microscope. The soil contained approximately 5 AMF spores per 100 g soil. For Expt 2, the soil was collected from the same rice field as for Expt 1; however, spore abundance then had doubled to around 10 AMF spores per 100 g soil. Overall, spore density was low, likely because the rice field is flooded during the rice-growing season. We used unsterilized soil in both experiments; therefore, indigenous AMF could colonize the rice roots. As AMF spore density in the soil was low, we inoculated the soil with AMF spores for the high-AMF colonization treatment. We used 3 g of commercial granule AMF inoculum (RootGrow Professional, Kent, UK) which contained *Funneliformis mosseae*, *F. geosporus*, *Claroideoglomus claroideum*, *Glomus microaggregatum*, and *Rhizophagus irregularis* (Robinson Boyer et al. 2016) in the pots in the planting hole at 5 cm depth during transplanting. The spore density of the AMF inoculum was about 10 spores per g of inoculum.

### Planting, water, and nutrient treatments

Rice seedlings were grown for 14 days in plug trays before being transplanted to the pots. One seedling was transplanted to the center of each pot. Rice plants were well-watered during the first 42 days by watering the pots every 2 days. The soil water potential was above − 10 kPa in order to ensure AMF symbiosis establishment and healthy rice plants. The drought treatment was applied at 42 days after planting (DAP), the day we stopped watering the pots in the drought treatment. In Expt 1, the withholding of water in the drought treatment was continued until the soil water potential reached − 40 kPa. This treatment involved no watering for 4 days. After that, the soil in the pots was rewetted to 0 kPa, and then water was withheld again for 4 days. In Expt 2, the withholding of water in the drought treatment was continued until the soil water potential reached − 80 kPa. This treatment involved no watering for 6 days. After that, the soil in the pots was rewetted to 0 kPa, and then water was withheld again for 6 days. The drying and rewetting cycle was done repeatedly until 90 DAP in both experiments. After 90 DAP, the pots remained well-watered, allowing the plants to recover in order to assess final yield and mycorrhizal root colonization. In the well-watered treatment, the soil water potential was maintained at around 0 to − 10 kPa throughout both experiments. Our experiment can thus be classified as type II (drying–rewetting cycle type) in the subdivision of He and Dijkstra (2014). We applied nitrogen (N) fertilizer at the rate of 20 kg N ha^−1^ to all pots 30 DAP. Later, we applied fertilizer which contained N-P_2_O_5_-K_2_O at the rate of 20–20-10 kg ha^−1^ when the rice plants were 60 DAP. The fertilizer was dissolved and added with irrigation water. Harvesting was done after the grain was fully ripened. The harvesting date depended on the physiological age and was somewhat different for each variety (Supplementary table 2). In all experiments, the harvesting date of the rice plants in the drought treatment was about 2 weeks later than in well-watered treatment. The timeline of the experiment is depicted in supplementary Fig.1.

### AMF colonization

After harvest, fresh roots were collected, washed, weighed, and a subsample of approximately 10% taken for quantifying AMF colonization. Roots were stained by clearing in boiling 2.5% KOH solution at 90 °C for 10 min, then left in 1% HCl solution overnight (Koske and Gemma 1989). Finally, they were stained with 0.05% Trypan blue solution (dissolved in glycerin). AMF colonization was quantified by counting vesicles, arbuscules, and intraradical hyphae at × 400 magnification according to Giovannetti and Mosse (1980). The results were expressed as percentage of root length colonized (RLC).

### Rice dry biomass (shoot, root, grain)

Rice was harvested when the grains were fully ripened. We harvested shoots, roots, and grain separately. We first harvested the grain by using scissors to cut and separate the panicles from the shoots. We separated filled and unfilled grain from the panicles and assessed the dry weight of filled grain. The shoots were harvested at about 5 cm above the ground. We recorded shoot fresh weight, and then dried the shoots at 80 °C for 48 h to assess dry weight. Roots were collected by washing the roots and subsequent careful removal of soil material and fragments of organic matter in fresh water. The fresh weight of the roots was recorded. Approximately 10% of the roots were taken for AMF colonization measurement, and the rest were dried at 80 °C for 48 h to assess dry weight.

### Stomatal conductance and chlorophyll fluorescence

We measured stomatal conductance (*g*_*s*_) and chlorophyll fluorescence (PS II efficiency) of the rice plants. The measurements were done 54 DAP, hence 12 days after the initiation of the drought treatment. For both measurements, we chose the three youngest fully emerged leaves from each plant and measured at the middle part of the leaf for all selected leaves. Stomatal conductance was measured from 9.30 to 12.00 am by steady AP4 porometer (Delta-T devices, UK). Chlorophyll fluorescence was measured by chlorophyll fluorometer (MINI-PAM, WALZ, Germany). The results of minimum (*F*_0_) and maximum (*F*_*m*_) fluorescence that the plant leaf can absorb were recorded to quantify the maximum quantum efficiency of photosystem II (PSII) photochemistry (*F*_*v*_/*F*_*m*_), with *F*_*v*_ = *F*_*m*_-*F*_0_ (Murchie and Lawson 2013).

### Nitrogen and phosphorus concentration

For the analysis of nutrient concentrations, the third leaf from the apex was chosen. The leaf samples were collected during the panicle development stage. We collected the leaves after the rice was flowering (90 DAP), but in Expt 2, we collected the leaves before the rice was flowering (80 DAP). Five leaves were collected from each pot and dried at 80 °C for 72 h before grinding. The ground plant materials were submitted to the laboratory at Khon Kaen University for N and P analysis. N concentration was quantified by the Kjeldahl method (Bremner 1965), and P concentration was analyzed by wet digestion (nitric-perchloric digestion) and spectrophotometry (Land Development Department (Thailand) 2011). The N:P ratio (based on concentrations) was calculated to assess to what extent plants were limited by N and/or P (Güsewell 2004).

### Plant hormone analysis

We analyzed ABA and indole-3-acetic acid (IAA) in Expt 2. The leaves of the rice plants were collected at 48 DAP, hence 6 days after applying drought. From each pot, we collected the second leaf from the apex, three leaves per pot. The leaves were cut, wrapped in aluminum foil, and put directly into liquid nitrogen. The leaf samples were ground in liquid nitrogen and stored in Eppendorf tubes at − 80 °C. The frozen samples were shipped with dry ice from Thailand to the Netherlands for analyses. ABA and IAA were analyzed at the Plant Physiology Laboratory, Wageningen University, the Netherlands. The extraction and analyses of ABA and IAA were done as described in Kolachevskaya et al. (2017) except that the weight of the samples was 4 mg DW.

### Statistical analyses

Data are presented as means ± standard error. The results were analyzed in SPSS version 20. Data were first checked for normality (Shapiro-Wilk) and homogeneity of variance (Levene’s test). Non-homogeneously distributed data such as N and P concentrations were log-transformed before analysis. Expt 1 and 2 were analyzed separately. Three-way ANOVA was used to determine significant sources of variation at *P* < 0.05. We used Tukey’s honestly significant difference test to determine significant differences among treatments.

## Results

### AMF colonization

**Fig. 1:**
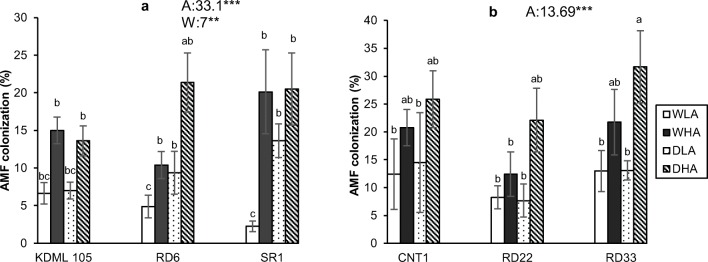
Fractional AMF colonization in rice roots of six different rice varieties in relation to drought and AMF inoculation. **a** Experiment 1, rice varieties KDML 105, RD6, SR1; **b** experiment 2, rice varieties CNT1, RD22, RD33. WLA, well-watered and low AMF; WHA, well-watered and high AMF; DLA, drought and low AMF; and DHA, drought and high AMF. The error bar is related to SE. Bars topped by the same letter are not significantly different by Tukey HSD (*P* < 0.05). The significant effects of mycorrhiza (A), water (W), and variety (V), and their interactions are presented as the *F* value with test of significance (*P < 0.05; **P < 0.01; ***P < 0.001) of three-way ANOVA. Non-significant factors are not shown (see Supplementary Table 3 and Table 4 for more information)

In Expt 1, AMF inoculation and water availability were significant sources of variation, whereas rice variety and all two-way and three-way interactions were not. AMF inoculation resulted in significantly higher AMF colonization compared to the low-AMF colonization treatment (Fig. 1a). Average root length colonization (RLC) was 7 + 1.6% in the low-AMF colonization (i.e., non-inoculated) treatments, and 16 + 3.3% in the high-AMF colonization (i.e., inoculated) treatments (Fig. 1a). Root colonization was higher in plants growing in the soil that was subjected to drought than in plants growing under well-watered conditions (Fig. 1a). There was no significant difference in AMF colonization between KDML 105, RD6, and SR1 varieties.

In Expt 2, AMF inoculation was the only significant source of variation. Colonization was significantly higher in the high-AMF colonization treatment (22 ± 5.1%) than in the low-AMF colonization treatment (11 ± 5.3%; Fig. 1b).

### Shoot and root dry biomass

**Fig. 2:**
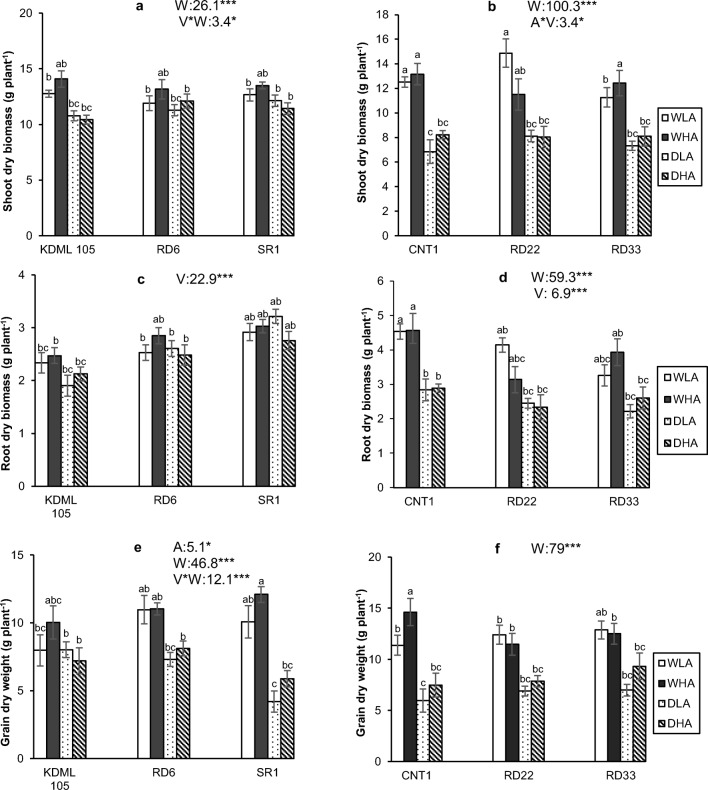
The shoot and root biomass and grain yield of six rice varieties in relation to drought and AMF inoculation. **a**, **c**, **e** Experiment 1 and **b**, **d**, **f** experiment 2. WLA, well-watered and low AMF; WHA, well-watered and high AMF; DLA, drought and low AMF; and DHA, drought and high AMF. The error bar is related to SE. Bars topped by the same letter are not significantly different by Tukey HSD (*P* < 0.05). The significant effects of mycorrhiza (A), water (W), and variety (V), and their interactions are presented as the *F* value with test of significance (*P < 0.05; **P < 0.01; ***P < 0.001) of three-way ANOVA. Non-significant factors are not shown (see Supplementary Table 3 and Table 4 for more information)

In Expt 1, water availability and the interaction between water availability and rice variety were significant sources of variation for shoot dry biomass, whereas the other factors and interactions were not. Shoot dry biomass was significantly greater in well-watered conditions compared to drought (Fig. 2a). Average shoot dry biomass decreased from 13.02 + 0.31 g in well-watered conditions to 11.36 + 0.28 g in the drought treatment (Fig. 2a). The negative effect of drought tended to be stronger in the drought-tolerant variety KDML 105 than in the drought-sensitive variety RD6. For root dry biomass, only rice variety was a significant source of variation, whereas the other main factors and all interactions were not. The drought-tolerant variety SR1 had highest root dry biomass, whereas the equally drought-tolerant KDML 105 had the lowest. Drought had no effect on root dry biomass (Fig. 2c).

For grain yield in Expt 1, AMF and water availability were significant sources of variation. The rice variety × water availability interaction also was significant, whereas rice variety and the other interactions were not (Fig. 2e). Grain yield was reduced under drought, especially for the drought-tolerant SR1, but hardly so for the equally drought-tolerant KDML 105. Mycorrhiza had a positive effect on grain yield (Fig. 2e).

The effect of drought on plant biomass in Expt 2 was much stronger than in Expt 1, because the soil was dried for 6 rather than 4 days, and soil water potential decreased to − 80 kPa, rather than − 40 kPa. For shoot dry biomass in Expt 2, water was a significant source of variation. The interaction between AMF and rice variety also was a significant source of variation, whereas both factors alone and the other interactions were not. Drought significantly reduced shoot dry biomass in all treatments. The shoot dry biomass decreased from 12.62 + 0.92 g in the well-watered treatments to 7.78 + 0.63 g in the drought treatments (Fig. 2b). For root dry biomass, both water availability and rice variety were significant sources of variation, whereas the mycorrhiza × variety interaction was marginally significant (*P* = 0.051; Supplementary table 4). Mycorrhiza and the other interactions were not significant sources of variation. Drought reduced root dry biomass, and the effect was strongest for the drought-tolerant CNT1 (Fig. 2d).

In Expt 2, water was again a significant source of variation for grain yield, whereas the effect of AMF was marginally significant (*P* = 0.054; Supplementary Table 4). Drought very substantially (more than 40%) reduced grain yield (Fig. 2f).

### Stomatal conductance and chlorophyll fluorescence

**Fig. 3:**
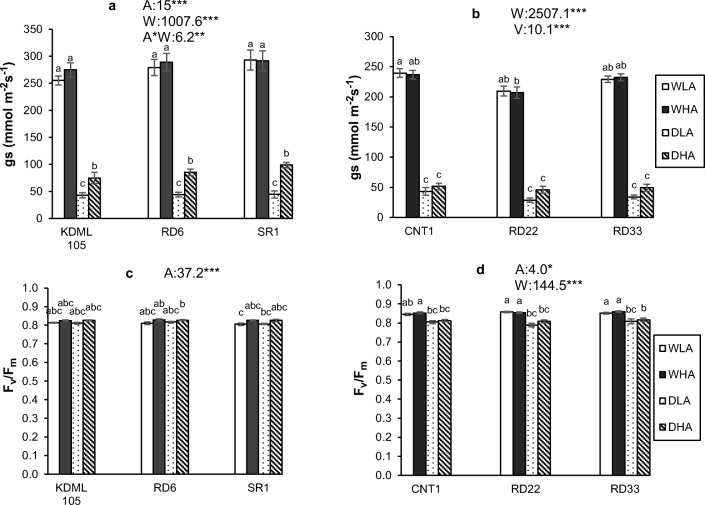
The stomatal conductance (*g*_*s*_) and the maximum quantum efficiency of PS II photochemistry (*F*_*v*_/*F*_*m*_). **a**, **c** Experiment 1 and **b**, **d** experiment 2. WLA, well-watered and low AMF; WHA, well-watered and high AMF; DLA, drought and low AMF; and DHA, drought and high AMF. The error bar is related to SE. Bars topped by the same letter are not significantly different by Tukey HSD (*P* < 0.05). The significant effects of mycorrhiza (A), water (W), and variety (V), and their interactions are presented as the *F* value with test of significance (*P < 0.05; **P < 0.01; ***P < 0.001) of three-way ANOVA. Non-significant factors are not shown (see Supplementary Table 3 and Table 4 for more information)

In Expt 1, water availability and AMF as well as their interaction exerted a significant effect on stomatal conductance (Fig. 3a). Drought consistently and very strongly decreased stomatal conductance by more than 75% compared to well-watered treatments, showing stomatal closure during drought. Plants with higher AMF colonization levels exhibited higher stomatal conductance than plants with lower AMF colonization levels, and this effect was much stronger under drought than under well-watered conditions (Fig. 3a). Rice variety had only a marginal effect on stomatal conductance (*P* = 0.063; Supplementary Table 3). Stomatal conductance tended to be lower in the drought-tolerant KDML 105 than in the two other rice varieties. For maximum quantum efficiency of PS II in Expt 1, only AMF inoculation was a significant source of variation, whereas the other main factors and all interactions were not (Fig. 3c). Plants with higher AMF colonization exhibited higher values of *F*_*v*_/*F*_*m*,_ even though the difference was relatively small.

In Expt 2, water availability was again the main source of variation for stomatal conductance (Fig. 3b). Mycorrhiza now became a marginally significant source of variation, whereas rice variety was a significant source. Drought again severely reduced stomatal conductance by 80%. Stomatal conductance was significantly lower in leaves of the rice variety RD22 than in leaves of CNT1 and RD33. In this experiment, AMF was again a significant source of variation for *F*_*v*_/*F*_*m*_. When soil water potential was reduced to − 80 kPa (rather than − 40 kPa as in Expt 1), water also was a significant source of variation. Drought significantly reduced *F*_*v*_/*F*_*m*_, whereas plants with higher AMF colonization levels still exhibited higher *F*_*v*_/*F*_*m*_ than plants with lower AMF colonization levels (Fig. 3d).

### Nitrogen and phosphorus concentrations

**Fig. 4:**
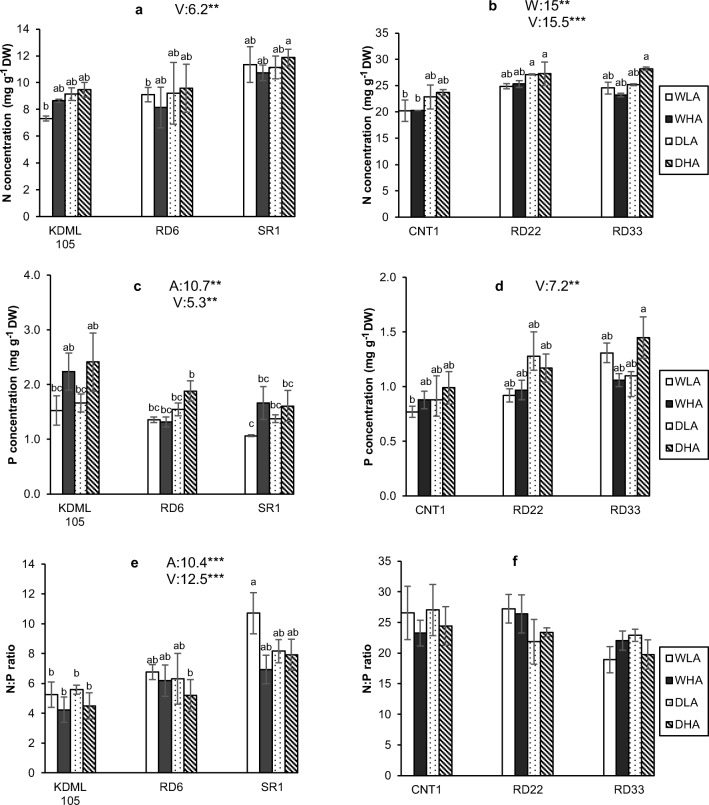
N, P concentrations in rice leave and N:P ratio. **a**, **c**, **e** Experiment 1 and **b**, **d**, **f** experiment 2. WLA, well-watered and low AMF; WHA, well-watered and high AMF; DLA, drought and low AMF; and DHA, drought and high AMF. The error bar is related to SE. Bars topped by the same letter are not significantly different by Tukey HSD (*P* < 0.05). The significant effects of mycorrhiza (A), water (W), and variety (V) and their interactions are presented as the *F* value with test of significance (*P < 0.05; **P < 0.01; ***P < 0.001) of three-way ANOVA. Non-significant factors are not shown (see Supplementary Table 3 and Table 4 for more information)

In Expt 1, variety was a significant source for variation for both N and P. For P mycorrhiza was also a significant source of variation. The other factors and their interactions were not significant sources of variation (Supplementary table 3). N and P concentrations were highest in KDML 105 and lowest in SR1. Mycorrhiza enhanced P concentration in the leaves of the three varieties. N:P ratios were lowest in KDML 105 and highest in SR1, with higher AMF colonization plants having a lower N:P ratio. N:P ratios were almost always below 10 (except for one treatment where N:P ratio was 11), suggesting that plants were N-limited, whereas the slightly lower N:P ratio in high-colonization treatments indicated either luxury P uptake or more severe N limitation (Fig. 4a, c, e). The low N concentrations (lower than 10 mg / g) also provided evidence of N-limitation under almost all growing conditions.

In Expt 2, variety again was a significant source of variation for both N and P. Water availability was a significant source of variation for N, whereas it was marginally significant for P (*P* = 0.056; Supplementary table 4; Fig. 4b, d, f). Mycorrhiza was not a significant source of variation for N or P, nor was any interactions. N concentrations were much higher in Expt. 2 than in Expt. 1, whereas P concentrations were much lower. This resulted in much higher N:P ratios of above 20, indicative of P limitation. CNT1 had significantly lower N and P concentrations than the other two varieties. However, the differences in N concentrations between varieties were smaller than differences in P concentrations, and CNT1 had the highest N:P ratio. Drought increased N concentrations and tended to increase P concentrations.

### Plant hormones (ABA and IAA)

**Fig. 5:**
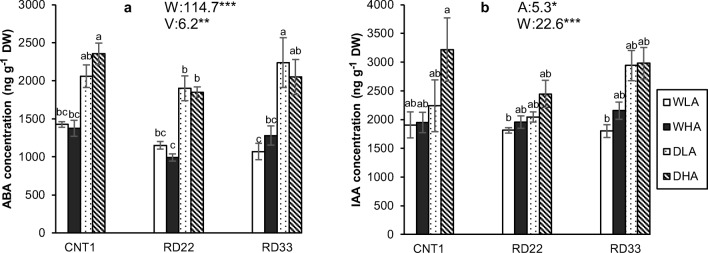
Abscisic acid (ABA) and indole-3-acetic acid (IAA) hormones concentration in rice leaves. WLA, well-watered and low AMF; WHA, well-watered and high AMF; DLA, drought and low AMF; and DHA, drought and high AMF. The error bar is related to SE. Bars topped by the same letter are not significantly different by Tukey HSD (*P* < 0.05). The significant effects of mycorrhiza (A), water (W) and variety (V), and their interactions are presented as the *F* value with test of significance (*P < 0.05; **P < 0.01; ***P < 0.001) of three-way ANOVA. Non-significant factors are not shown (see Supplementary Table 3 and Table 4 for more information)

The concentrations of ABA in leaves were significantly different among rice varieties (Fig. 5a). Rice variety CNT1 had higher ABA level in leaves than RD22 and RD33 varieties. In all varieties, ABA was significantly higher in plants grown under drought than under well-watered conditions (Fig. 5a). Drought increased ABA approximately by 70% compared to the well-watered treatments. The different levels of AMF colonization did not show significant differences in ABA concentration, although higher AMF colonization tended to decrease ABA in the rice varieties RD22 and RD33 under drought.

Drought significantly increased IAA in all three rice varieties (Fig. 5b). The increase of IAA was more than 35% higher under drought than in well-watered conditions (Fig. 5b). Plants with higher AMF colonization showed a significantly higher content of IAA in leaves than plants with lower AMF colonization (Fig. 5b).

## Discussion

Addition of commercial inoculum increased AMF colonization, possibly through both changes in inoculum potential and changes in AMF species composition. We did not assess species composition of the low- and high-AMF treatments, so cannot evaluate the relative importance of possible qualitative changes in the AMF community. Our study showed that inoculum addition resulted in higher grain yields (Expt 1; marginally so in Expt 2), whereas there were no effects on shoot and root dry biomass. There also were no significant mycorrhiza × water availability interactions, except for root dry biomass in Expt. 2, partly supporting our first hypothesis. The second hypothesis also was partly supported by a significant effect of mycorrhizas and the mycorrhiza × water availability interaction for stomatal conductance (Expt 1; marginally so for Expt 2); quantum efficiency was significantly influenced by mycorrhizas in both experiments, but the mycorrhiza × water availability interaction was not significant. There was no effect of mycorrhizas on ABA, whereas the mycorrhizal effect on IAA was significant. The results therefore confirmed our hypotheses on the positive effects of increased AMF colonization on rice under drought, but an interaction between AMF and drought was seldom evident.

### AMF colonization

Our experiments showed similar fractional colonization as previous studies. Zhang et al. (2014) reported 2–3% RLC in non-inoculated rice plants and 12–19% RLC in rice roots inoculated with AMF. Wangiyana et al. (2006) found 3–5% RLC in rice roots growing in paddies without AMF inoculation. AMF inoculation increased RLC, suggesting inoculum limitation in the field. The higher root colonization in Expt 2 than in Expt 1 is likely due to the build-up of AMF in the soil between years, as inoculum potential doubled from five to ten AMF spores per 100 g soil between samplings. However, evidence for inoculum limitation, as shown by higher grain yields of plants and higher stomatal conductance and chlorophyll fluorescence with higher levels of colonization, persisted in the second year. Drought increased mycorrhizal colonization significantly in Expt 1 (but not in Expt 2), in agreement with studies by Lumini et al. (2011) and Vallino et al. (2014), who observed that mycorrhizal colonization was higher in rice roots growing in dry conditions compared to submerged conditions. These studies reflected a change from anaerobic to aerobic conditions and considering the strictly aerobic characteristics of AMF such an increase is not surprising. It is not clear whether our well-watered treatment (0 kPa) created anaerobic conditions. As the effect of drought on mycorrhizal colonization is much smaller than that of the inoculation treatment, our results suggest that a shift from irrigated rice to rain-fed rice will only gradually result in increases in mycorrhizal inoculum potential, and that inoculum addition or management, possibly with the help of cover crops when fields are fallow, could contribute to the build-up and maintenance of sufficient mycorrhizal inoculum, which then has beneficial consequences for yield.

### Shoot and root dry biomass and grain yield

Drought reduced rice shoot dry biomass. The moderate drought of Expt 1 had a smaller impact on root dry biomass than the more severe drought of Expt 2. The decrease of root and shoot dry biomass under drought could be both due to the reduced availability of water, which impeded photosynthetic carbon gain, and through drought-induced reduction of nutrient availability, especially of nutrients with low mobility such as P (Prasertsak and Fukai 1997; Suriyagoda et al. 2014). The responses of rice roots under drought contradicted previous reports, which proposed that rice develops increased roots under drought (Yoshida and Hasegawa 1982). Our results did not show that the most drought-tolerant varieties produced more roots than the less drought-tolerant varieties. The marginally significant interaction between mycorrhizas and water in Expt 1 (*P* = 0.064) suggests that under moderate, but not severe drought, AMF can somewhat alleviate this interaction effect.

Drought decreased grain yield of rice in both experiments. Moderate drought of 4 days had a smaller negative effect than strong drought that lasted 6 days. Our result is in line with the study of Venuprasad et al. (2007), who found a reduction of rice yield of more than 60% under drought. Similarly, Ghosh and Singh (2010) found significantly decreased rice yield when soil water potential decreased to − 60 kPa. Both water and nutrients are major limiting factors for rice at the grain filling stage (Fageria 2003). Even though AMF did not increase shoot and root dry biomass, AMF increased rice grain yield in our experiments. In addition, Zhang et al. (2016) stated that AMF plants increase the allocation of N and P to rice panicles compared to non-AMF plants during the grain filling stage, and the grain yield of rice increased about 28%.

### Stomatal conductance and chlorophyll fluorescence

Drought substantially decreased stomatal conductance, which can be explained by the plant physiological mechanisms that enabled reduced water loss. When water becomes limiting, stomata are closed to prevent the loss of water via transpiration, which simultaneously reduces the exchange of CO_2_. As stated by Hasegawa and Yoshida (1982), the decrease in transpiration rate of upland rice occurs when soil water potential is reduced below − 20 kPa. Under drought, plants with higher AMF colonization exhibited higher stomatal conductance than plants with lower AMF colonization. With more severe drought in Expt 2, the effect was smaller with only a marginally significant effect. The meta-analysis of Augé et al. (2015) showed that, averaged over all studies, stomatal conductance of AMF plants is 24% higher than in non-AMF plants. Higher stomatal conductance might be due to the extension of hyphae to the water and nutrient sources that are inaccessible to plant roots (Smith and Read 2010), but has been hypothesized also to be due to hormonal changes consequent upon mycorrhizal colonization (Birhane et al. 2012). The interaction between AMF and drought in both experiments indicates that AMF can alleviate the negative consequences of stomatal closure under drought. This is in agreement with the meta-analysis of Worchel et al. (2013), who also found greater effects of AMF on the growth of grasses grown under dry than under normal conditions. Li et al. (2014) found a positive effect of AMF (*Rhizophagus intraradices*) on barley, but no effect of drought on stomatal conductance.

Quantum efficiency of PS II was unaffected by the moderate drought of Expt 1, but was significantly reduced by the strong drought of Expt 2. These results are in agreement with the study of Puteh et al. (2013), who reported that the quantum efficiency of rice decreased from 0.78 to 0.60 after 8 days of drought. In our Expt 1, where we did not provide water for 4 days, drought might not have been sufficiently severe to cause a reduction of quantum efficiency. Apparently, chlorophyll fluorescence acts at a different temporal scale than stomatal conductance, in that chlorophyll fluorescence is more resistant to a short drought (Trueba et al. 2019). The values of chlorophyll fluorescence in well-watered plants are close to the theoretical optimum of 0.83 (Björkman and Demmig 1987). Despite values of well-watered plants close to that theoretical maximum, plants with higher AMF colonization had slightly but significantly higher values of chlorophyll fluorescence, possibly due to sink stimulation of photosynthesis by the AMF symbiosis (Kaschuk et al. 2009). Beneficial effects of AMF on chlorophyll fluorescence also have been reported by de Andrade et al. (2015), who found that mycorrhizal rice plants had higher chlorophyll fluorescence under arsenate and arsenite pollution than non-mycorrhizal plants. Mathur et al. (2019), studying the response of wheat to very severe drought, also noted a beneficial effects of AMF on chlorophyll fluorescence. However, Porce et al. (2015) observed that mycorrhizal plants exhibited lower chlorophyll fluorescence than non-mycorrhizal plants, except at high salt levels where fluorescence values increased in mycorrhizal plants compared to a treatment with lower salt levels.

### Nutrient uptake: N and P concentration, N:P ratio

Drought limits the availability of nutrients for plant uptake (Suriyagoda et al. 2014). Drought effects were noted for plant biomass, but less so for nutrient concentrations. Mild drought of Expt 1 did not affect leaf N and P concentrations, whereas the more severe drought of Expt 2 did positively affect concentrations of both nutrients. Our results contrast with previous studies that reported reduction of P uptake under drought (Suriyagoda et al. 2014; Sardans and Peñuelas 2004). A meta-analysis by He and Dijkstra (2014) indicated that drought on average reduced plant N and P concentrations by 3.7 and 9.2% respectively. However, their meta-analysis also showed that drought experiments involving a drying–rewetting cycle (their type II, as in our experiment) did not have a negative effect on those concentrations, with rather non-significant positive effect sizes as in our experiment. Higher AMF colonization did not affect leaf N concentration, suggesting that N immobilization in the mycorrhizal mycelium was not important. N is important for grain filling and ripening. More than 60% of N is finally translocated from shoot to grain during the reproductive stage (Fageria 2003). AMF rice plants allocate more N to the panicle than non-AMF rice plants (Zhang et al. 2016). However, we cannot confirm the effects of AMF on N allocation, because we did not analyze the N concentration in the roots and grain in our experiments. Increasing mycorrhizal colonization increased P concentrations in Expt 1 but did not have an effect in Expt 2. In Expt 1, rice plants were N- rather than P-limited (to judge from N:P ratios below 10), so a major part of the additionally acquired P could not be translated into biomass increase but rather showed as higher P concentrations. In Expt 2, plants were P-limited, as N:P ratios were above 20, but it is unclear why plants did not show a biomass response to higher AMF colonization.

### Plant hormones (ABA and IAA)

ABA has been considered the abiotic stress hormone by Bahadur et al. (2019), and the increase of ABA under drought agrees with their review. Dobra et al. (2010) found that ABA increased about 50–80 times in tobacco leaves grown under drought. Ludwig-Müller (2010) and Bahadur et al. (2019) also included data on changes in ABA levels due to mycorrhizal colonization, and both decreases in root ABA (in tomato and the legume (*Glycyrrhiza*) and increases in ABA (in maize) have been noted. Reduction of ABA in AMF plants has also been reported by Estrada-Luna and Davies (2003) in mycorrhizal *Capsicum annuum* compared to non-inoculated plants. Higher levels of ABA would result in stomatal closure and a lowering of stomatal conductance, so we cannot explain a mycorrhiza effect on stomatal conductance without an effect on ABA levels in our study. According to Borghi et al. (2015), plants may produce or transport more ABA to the leaves to regulate stomatal closure when subjected to drought. However, earlier studies referred to ABA levels in roots, and therefore may not be comparable to our study where we assessed ABA levels in shoots as data on root–shoot signaling through hormones remain scarce (Ludwig-Müller 2010).

Both drought and AMF resulted in increased IAA levels. The increase of IAA in response to drought is not consistent with observations by Dobra et al. (2010) on tobacco, who observed decreases in IAA levels in young leaves (we collected the second leaf for hormone analysis), but increases in IAA levels in middle and lower leaves and also in roots. Literature data indicate both cases where IAA levels were upregulated by AMF and cases where hormone levels were unchanged, but most of the available data refer to changes in hormone levels in roots (Ludwig-Müller 2010). Some plant species produce IAA also to stimulate the symbiosis with soil micro-organisms under stress conditions. That could be the reason that IAA content in plants with higher AMF colonization was higher than in plants with lower AMF colonization. AMF inoculation also may increase the level of IAA in plant leaves. Fitze et al. (2005) found an increase of IAA in maize after 20 and 30 days after AMF inoculation. However, there are other studies that reported that IAA does not change with AMF inoculation (Torelli et al. 2000; Shaul-Keinan et al. 2002).

## Conclusion

AMF colonization in rice fields is usually low, but it may be possible to enhance colonization by adding AMF inoculum. Increased RLC improves rice plant performance through uptake of nutrients such as N and P, resulting in higher grain yields (hence a higher harvest index), without much effect on total plant biomass. Moreover, AMF increase photosynthesis, especially under drought, via a smaller reduction in stomatal closure and by maintaining higher levels of chlorophyll fluorescence. These effects are likely mediated both through nutrients and through regulation of plant hormones, especially IAA. AMF therefore contribute to a better recovery after drought resulting in higher rice grain yields. The outcomes of our study may be relevant under climate change where drought is becoming a major factor restricting rice production. Increasing AMF colonization may be important for water savings.

## Electronic supplementary material

ESM 1 (DOCX 46.4 kb)
