# Supplementary material for: Increased arbuscular mycorrhizal fungal colonization reduces yield loss of rice (Oryza sativa L.) under drought
Source: Mycorrhiza. 2020 Apr 15;30(2):315–28. doi: 10.1007/s00572-020-00953-z (PMC7228911; doi:10.1007/s00572-020-00953-z)
Supplement: Supplementary file 1 — (DOCX 46.4 kb) [file 572_2020_953_MOESM1_ESM.docx]

**Supplementary figures and tables**

Supplementary figure 1: Time line of the experiment 1 and 2.

Supplementary table 1: Properties of the soil used in expt 1 and expt 2

| Properties | Experiment 1 | Experiment 2 |
| --- | --- | --- |
| Soil texture | Sandy loam | Sandy loam |
| Bulk density (g cm^-3^) | 1.3 | 1.4 |
| Water holding capacity (%) | 25.5 | 30.6 |
| pH (1:1 H_2_O) | 7.5 | 7.3 |
| Organic matter (%) | 0.62 | 0.57 |
| Total N (mg/kg) | 270 | 310 |
| Extractable P (Bray II method, mg/kg) | 19.5 | 23.5 |
| Total K (mg/kg) | 995 | 964 |

Supplementary table 2: Rice varieties used in both experiments.

(Source: <http://kkn-rsc.ricethailand.go.th/index.php/e-library/varieties>)

|  | Experiment 1 | | | Experiment 2 | | |
| --- | --- | --- | --- | --- | --- | --- |
|  | KDML 105 | RD6 | SR1 | CNT1 | RD22 | RD33 |
| *Photoperiod sensitivity* | Yes | Yes | No | No | No | No |
| *Growing period* | May-November | May - November | Year round | Year round | Year round | Year round |
| *Harvesting (days after sowing)* | 25-Nov* | 21-Nov* | 138 | 130 | 130 | 130 |
| *Average grain yield (kg/ ha)* | 2251 | 4129 | 4452 | 4588 | 4241 | 3057 |
| *Average height (cm)* | 140 | 154 | 122 | 113 | 120 | ** |
| *Drought tolerance* | High | Moderate | High | High | Low | High |

* *The estimated harvesting date (because the harvest of photoperiod-sensitive varieties depends on day length)*

** *No data*

Supplementary table 3: Analysis of Variance showing the main and interaction effects of AMF, water and variety treatments on rice growth in Experiment 1. RLC = fractional root length colonization; SDW=Shoot dry weight, RDW = Root dry weight, GDW = Grain dry weight, F_v_/F_m_ = The maximum quantum efficiency of PS II photochemistry, g_s_ = Stomatal conductance, N = Nitrogen and P=Phosphorus mass-fraction. Bold numbers indicate significant results at P <0.05.

| Independent variables |  | RLC | | SDW | | RDW | | GDW | | F_v_/F_m_ | | g_s_ | | N mass-fraction | | P mass-fraction | | N:P ratio | |
| --- | --- | --- | --- | --- | --- | --- | --- | --- | --- | --- | --- | --- | --- | --- | --- | --- | --- | --- | --- |
|  | df | F | P-value | F | P-value | F | P-value | F | P-value | F | P-value | F | P-value | F | P-value | F | P-value | F | P-value |
| AMF (A) | 1 | 33.1 | **<0.001** | 2.6 | 0.109 | 0.2 | 0.698 | 5.1 | **0.027** | 37.2 | **<0.001** | 15 | **<0.001** | 0.2 | 0.675 | 10.7 | **0.002** | 10.4 | **<0.001** |
| Water (W) | 1 | 7.0 | **0.010** | 26.1 | **<0.001** | 3.5 | 0.066 | 46.8 | **<0.001** | 0.1 | 0.785 | 1007.6 | **<0.001** | 1.4 | 0.241 | 2.4 | 0.130 | 0.1 | 0.799 |
| Variety (V) | 2 | 1.7 | 0.197 | 0.6 | 0.545 | 22.9 | **<0.001** | 1.5 | 0.236 | 1.1 | 0.343 | 2.9 | 0.063 | 6.2 | **0.005** | 5.3 | **0.009** | 12.5 | **<0.001** |
| A*W | 1 | 0.4 | 0.531 | 3.5 | 0.064 | 2.7 | 0.102 | 0.2 | 0.619 | 0.2 | 0.642 | 6.2 | **0.015** | 0.1 | 0.750 | 0.03 | 0.854 | 1.7 | 0.191 |
| A*V | 2 | 0.8 | 0.463 | 0.8 | 0.471 | 1.3 | 0.270 | 1.4 | 0.262 | 0.7 | 0.518 | 0.0 | 0.998 | 0.2 | 0.834 | 1.0 | 0.379 | 0.6 | 0.665 |
| V*W | 2 | 2.3 | 0.110 | 3.4 | **0.038** | 1.6 | 0.209 | 12.1 | **<0.001** | 0.2 | 0.854 | 0.5 | 0.613 | 0.2 | 0.836 | 0.3 | 0.736 | 0.1 | 0.909 |
| A*V*W | 2 | 2.3 | 0.104 | 0.4 | 0.684 | 1.2 | 0.311 | 0.4 | 0.677 | 0.4 | 0.670 | 0.9 | 0.430 | 0.3 | 0.736 | 0.3 | 0.723 | 0.6 | 0.649 |

Supplementary table 4: Analysis of Variance showing the main and interaction effects of AMF, water and variety treatments in Experiment 2. RLC = fractional root length colonization SDW=Shoot dry weight, RDW = Root dry weight, GDW = Grain dry weight, F_v_/F_m_ = The maximum quantum efficiency of PS II photochemistry, g_s_ = Stomatal conductance, N = Nitrogen and P=Phosphorus mass-fraction. Bold numbers indicate significant results P <0.05.

| Independent variables |  | RLC | | SDW | | RDW | | GDW | | F_v_/F_m_ | | g_s_ | | N mass-fraction | | P mass-fraction | | N:P ratio | |
| --- | --- | --- | --- | --- | --- | --- | --- | --- | --- | --- | --- | --- | --- | --- | --- | --- | --- | --- | --- |
|  | df | F | P-value | F | P-value | F | P-value | F | P-value | F | P-value | F | P-value | F | P-value | F | P-value | F | P-value |
| AMF (A) | 1 | 13.6 | **<0.001** | 0.03 | 0.865 | 0.001 | 0.974 | 3.8 | 0.054 | 4.0 | **0.048** | 3.3 | 0.073 | 0.6 | 0.450 | 0.3 | 0.578 | 0.4 | 0.565 |
| Water (W) | 1 | 2.1 | 0.147 | 100.3 | **<0.001** | 59.3 | **<0.001** | 79 | **<0.001** | 144.5 | <**0.001** | 2507.6 | **<0.001** | 15.0 | **0.002** | 4.5 | 0.056 | 0.3 | 0.599 |
| Varieties (V) | 2 | 2.2 | 0.114 | 1.0 | 0.360 | 6.9 | **0.002** | 0.6 | 0.543 | 1.0 | 0.385 | 10.1 | **<0.001** | 15.5 | **<0.001** | 7.2 | **0.009** | 2.8 | 0.103 |
| A*W | 1 | 1.7 | 0.197 | 1.6 | 0.212 | 0.3 | 0.567 | 0.7 | 0.412 | 1.0 | 0.329 | 3.7 | 0.057 | 1.4 | 0.256 | 0.9 | 0.352 | 0.1 | 0.744 |
| A*V | 2 | 0.2 | 0.796 | 3.4 | **0.037** | 3.1 | 0.051 | 1.4 | 0.252 | 0.0 | 0.998 | 0.3 | 0.741 | 0.1 | 0.939 | 0.3 | 0.755 | 0.4 | 0.678 |
| V*W | 2 | 0.0 | 0.981 | 0.6 | 0.576 | 0.8 | 0.458 | 1.0 | 0.366 | 1.7 | 0.192 | 3.0 | 0.058 | 0.2 | 0.853 | 0.6 | 0.547 | 1.1 | 0.369 |
| A*V*W | 2 | 0.2 | 0.845 | 1.3 | 0.288 | 0.9 | 0.391 | 1.4 | 0.250 | 1.1 | 0.332 | 0.2 | 0.862 | 1.1 | 0.372 | 2.3 | 0.139 | 0.7 | 0.531 |

Supplementary table 5: Analysis of Variance showing the main and interaction effects of AMF, water and variety treatments on abscisic acid (ABA) and Indole-3-acetic acid (IAA) hormones in Experiment 2. Bold numbers indicate significant results P <0.05.

| Independent variables |  | ABA | | IAA | |
| --- | --- | --- | --- | --- | --- |
|  | df | F | P-value | F | P-value |
| AMF (A) | 1 | 0.0 | 0.916 | 5.3 | **0.027** |
| Water (W) | 1 | 114.7 | **<0.001** | 22.6 | **<0.001** |
| Varieties (V) | 2 | 6.2 | **0.005** | 2.3 | 0.118 |
| A*W | 1 | 0.1 | 0.831 | 0.6 | 0.459 |
| A*V | 2 | 0.8 | 0.452 | 0.3 | 0.753 |
| V*W | 2 | 0.7 | 0.498 | 1.3 | 0.300 |
| A*V*W | 2 | 1.9 | 0.168 | 1.5 | 0.245 |
